# The relationship of physical exercise and suicidal ideation among college students: a moderated chain mediation model

**DOI:** 10.3389/fpsyg.2025.1624998

**Published:** 2025-07-02

**Authors:** Jinrui Zhou, Feng Sun

**Affiliations:** College of Sports Science, Nantong University, Nantong, China

**Keywords:** physical exercise, perceived social support, meaning in life, suicidal ideation, college students

## Abstract

**Background:**

This study examines the impact of physical exercise on college students’ perceived social support, meaning in life, and suicidal ideation, and further investigates the mediating and sequential mediating roles of perceived social support and meaning in life in the association between physical exercise and suicidal ideation, additionally exploring the moderating role of gender.

**Methods:**

A total of 545 Chinese college students completed the Physical Activity Rating Scale-3 (PARS-3), Perceived Social Support Scale (PSSS), Meaning in Life Questionnaire (MLQ), and Self-rating Idea of Suicide Scale (SIOSS). A bias-corrected percentile bootstrap approach was applied to assess the sequential mediating effects of perceived social support and meaning in life on the relationship between physical exercise and suicidal ideation.

**Results:**

(1) Physical exercise and suicidal ideation were negatively correlated (*r* = −0.296, *p* < 0.01). A significant direct effect of physical exercise on suicidal ideation was also detected (*β* = −0.154, *t* = −7.317, *p* < 0.01). (2) Physical exercise positively predicted perceived social support (*β* = 0.218, *t* = 8.900, *p* < 0.01) and meaning in life (*β* = 0.131, *t* = 6.456, *p* < 0.01). In turn, perceived social support positively predicted meaning in life (*β* = 0.513, *t* = 15.429, *p* < 0.01) and negatively predicted suicidal ideation (*β* = −0.243, *t* = −6.286, *p* < 0.01). Furthermore, meaning in life exerted a negative effect on suicidal ideation (*β* = −0.246, *t* = −5.885, *p* < 0.01). (3) Both perceived social support and meaning in life significantly mediated the association between physical exercise and suicidal ideation via three pathways: physical exercise → perceived social support → suicidal ideation (mediating effect = −0.053); physical exercise → meaning in life → suicidal ideation (mediating effect = −0.032); and physical exercise → perceived social support → meaning in life → suicidal ideation (mediating effect = −0.028). (4) Gender moderated the effects of physical exercise on meaning in life (*β* = −0.136, *p* < 0.01) and on suicidal ideation (*β* = 0.142, *p* < 0.01).

**Conclusion:**

Physical exercise directly reduces suicidal ideation among college students and indirectly influences it through perceived social support and meaning in life, both as individual mediators and in a sequential pathway. Gender moderates both the initial segment of the physical exercise → meaning in life → suicidal ideation pathway and the direct effect on suicidal ideation.

## 1 Introduction

Suicide constitutes a critical public health concern, causing profound harm to individuals, families, and communities (Franklin et al., 2016). The World Health Organization (WHO) reports that suicide ranks as the fourth leading cause of death globally among people aged 15–29 (World Health Organization, 2019). In China, suicide has emerged as the foremost cause of unnatural death in the college student demographic, representing 47.2% of such fatalities (Yang and Li, 2015). Although research on suicide rates among Chinese undergraduates indicates a downward trend, the overall risk remains alarmingly high (Wu et al., 2018). Suicidal behavior unfolds in three successive stages—ideation, attempts, and completed suicide—with suicidal ideation serving as a pivotal risk factor for subsequent lethal self-harm (Groleger et al., 2003).

Suicidal ideation encompasses thoughts of self-harm or explicit planning of suicide absent overt suicidal behaviors (Klonsky et al., 2016). Its occurrence is shaped by psychological, social, and physiological determinants. Psychologically, heightened interpersonal sensitivity (Zhao et al., 2023), perceived discrimination (Yu et al., 2024), and elevated perceived stress (Wen et al., 2024a) have each been identified as positive predictors of suicidal ideation. Zhang X. et al. (2017) further showed that negative emotions such as depression and anxiety significantly forecast suicidal ideation 1 year later, with self-injurious behaviors partially mediating this link. Social influences—such as campus bullying (Hong et al., 2024), interpersonal rejection (Zhang et al., 2024), and adverse life events (Liu et al., 2024)—also markedly contribute to suicidal ideation. Recent findings by Jin et al. (2024) indicate that social trauma exerts both a direct positive effect on college students’ ideation and an indirect effect via psychological distress. Physiologically, impaired sleep quality remains a robust risk factor and predictor of suicidal ideation even when adjusting for psychological and social variables (Qian et al., 2017; Pigeon et al., 2012). Temporal fluctuations in suicidal ideation have also been documented (Wyder and Leo, 2007).

Physical exercise, as a socially embedded activity, exerts considerable influence on social processes (Huang et al., 2019). Evidence suggests that engaging in regular exercise enhances subjective well-being (Yasunaga et al., 2021), social trust (Zhang J. et al., 2022), life satisfaction (Yao et al., 2023), and perceived social fairness (Sui et al., 2024) through improvements in physical function and psychological health. In contrast, inadequate habitual exercise exacerbates negative affective states (Oliveira et al., 2019). Through organized physical activity, college students cultivate social networks, accrue social capital, and strengthen both interpersonal and broader societal trust (Inman et al., 2015). Although Ji et al. (2024) demonstrated that physical exercise negatively predicts suicidal ideation among individuals with disabilities, scant research has explored this relationship within the college student population. This knowledge gap leaves the mechanisms by which exercise may mitigate suicidal ideation in this group poorly understood.

In summary, given that suicidal ideation constitutes the most sensitive indicator of suicide risk, its assessment among college students—and the identification of its determinants—is essential for informing effective crisis intervention strategies and safeguarding student well-being. The present study examines the association between physical exercise and suicidal ideation in college students and investigates the mediating roles of perceived social support and meaning in life.

### 1.1 Physical exercise and suicidal ideation

Physical exercise encompasses bodily movements designed to enhance health, defined by specific parameters of intensity, frequency, and duration (Liu, 2020). The exercise affect theory posits that regular physical activity exerts therapeutic benefits on depression, anxiety, and stress, distinguished by high adherence, minimal adverse effects, and enduring efficacy (Hallgren et al., 2017). Individuals who maintain consistent exercise routines tend to exhibit greater life vitality, an optimistic outlook, stronger interpersonal relationships, and proactive coping with life’s challenges (Sun et al., 2023). Empirical findings demonstrate that structured sport-dance training bolsters college students’ adaptive coping strategies and promotes psychological well-being (Yang et al., 2020). Negative emotional states—such as anxiety, depression, distress, and loneliness—are significant predictors of suicidal ideation (Chang et al., 2018; Mackenzie et al., 2006). By reducing the incidence of depressive and anxiety disorders and alleviating psychological distress, physical exercise serves as a pivotal intervention to mitigate suicidal ideation and forestall suicidal behaviors (Zhang Y. Q. et al., 2022). Based on this theoretical rationale, Hypothesis 1 proposes that physical exercise will negatively predict suicidal ideation among college students.

### 1.2 The mediating role of perceived social support

Perceived social support denotes an individual’s cognitive appraisal of assistance available from others when confronting life demands (Howard et al., 2019). Self-determination theory holds that social contexts facilitate personal motivation and its internalization by satisfying three basic psychological needs: autonomy, relatedness, and competence. Given that physical exercise functions as both a social endeavor and a form of interpersonal engagement, it serves as a primary means to enhance perceived social support (Lu, 2001). Research has identified a significant positive correlation between college students’ exercise participation and perceived social support, with engagement increasing support perceptions (Chen et al., 2013; Wu et al., 2008). Moreover, perceived social support is an essential protective factor for mental health maintenance and suicidal ideation prevention among undergraduates (Li, 1998). High levels of social support uphold positive affect and psychological well-being (Gong, 1994) and buffer the adverse health consequences of stressful events, shielding individuals from stress-induced decline (Cohen and Wills, 1985). Findings show that students perceiving stronger support from family and institutions demonstrate significantly lower vulnerability to suicidal ideation (Miller et al., 2015). Based on this theoretical framework, Hypothesis 2 proposes that perceived social support mediates the relationship between physical exercise and suicidal ideation in college students.

### 1.3 The mediating role of meaning in life

Meaning in life refers to the extent to which individuals understand the significance of their existence and interpret their life purpose, mission, and central goals (Steger et al., 2006). In Chinese cultural contexts, adults derive existential meaning across five dimensions: social engagement, personal growth, relational harmony, life enjoyment, and physical–mental wellness (Tian et al., 2023). Empirical evidence shows a positive association between physical exercise and meaning in life, as regular activity presents controlled challenges that foster meaning perception and amplify existential significance (Ju, 2017). Flow experiences during exercise and endorphin release further enhance one’s sense of meaning (Ding and Fang, 2002). Concurrently, meaning in life is closely linked to psychological functioning: individuals who perceive their lives as meaningful exhibit adaptive outcomes such as reduced academic anxiety (Xu and Zhang, 2007), improved stress regulation (Li, 2006), and elevated life satisfaction (Fu et al., 2012). Conversely, a deficit in meaning in life correlates with heightened suicidal ideation, whereas cultivating existential purpose mitigates its severity (Jose and Angeina, 2019). Based on this theoretical synthesis, Hypothesis 3 posits that meaning in life mediates the relationship between physical exercise and suicidal ideation in college students.

### 1.4 The chain-mediating effect of perceived social support and meaning in life

According to self-determination theory, satisfying the need for social relatedness cultivates a heightened sense of meaning in life (Miller et al., 1988). Conversely, inadequate social support fosters maladaptive cognitive schemas marked by pessimistic evaluations of life trajectories and outcomes, thereby undermining well-being, life satisfaction, and existential purpose (Li, 2015). Empirical evidence indicates that strong perceived social support not only establishes the basis for enhanced meaning in life but also actively drives the search for existential significance (Zhang G. H. et al., 2017). Drawing upon this theoretical synthesis, Hypothesis 4 posits that perceived social support and meaning in life sequentially mediate the relationship between physical exercise and suicidal ideation among college students.

### 1.5 The moderating role of gender

Under the combined influence of societal gender norms, personality characteristics, and lifestyle habits, male and female college students display distinct patterns in interpersonal emotional experiences, daily routines, and engagement in physical exercise (Dong and Mao, 2018). It remains to be empirically determined whether gender moderates the pathways linking physical exercise, perceived social support, meaning in life, and suicidal ideation in this population. Accordingly, we propose Hypothesis 5: Gender moderates the relationships among these constructs, specifically: H5a: Gender moderates the effect of physical exercise on perceived social support; H5b: Gender moderates the effect of physical exercise on meaning in life; H5c: Gender moderates the effect of physical exercise on suicidal ideation; H5d: Gender moderates the effect of perceived social support on meaning in life; H5e: Gender moderates the effect of perceived social support on suicidal ideation; H5f: Gender moderates the effect of meaning in life on suicidal ideation.

**Figure 1:**
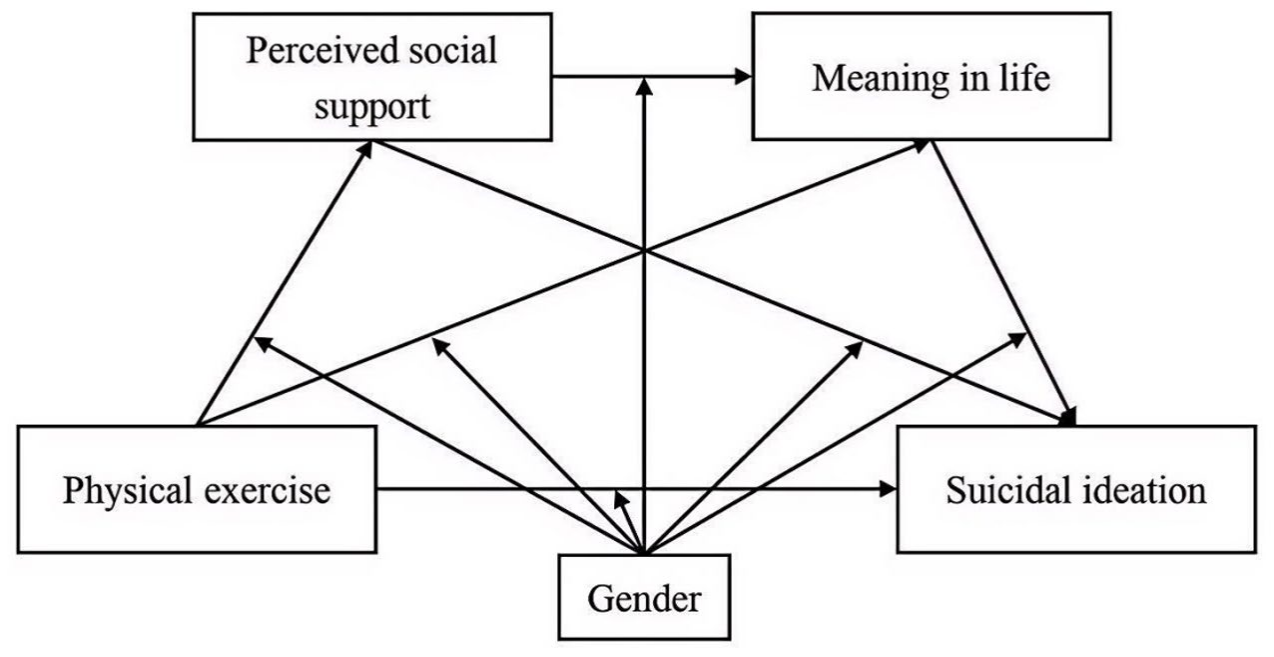
The hypothetical structure model.

Thus, this study advances the following conceptual framework (Figure 1): (1) To assess the direct effect of physical exercise on suicidal ideation among college students; (2) To investigate the mediating role of perceived social support in the association between physical exercise and suicidal ideation; (3) To examine the mediating effect of meaning in life in the link between physical exercise and suicidal ideation; (4) To explore the sequential mediating pathway through perceived social support and meaning in life; (5) To determine the moderating role of gender across these relationships.

## 2 Methods

### 2.1 Participants

This study employed a two-stage sampling strategy. First, universities in Jiangsu Province, China, were classified by geographic region and institutional type, followed by convenience sampling. The final cohort comprised undergraduates recruited from Soochow University, Nanjing Forestry University, and Nantong University. Participant selection was stratified by gender and academic year to improve representativeness. To ensure informed consent and voluntary participation, researchers outlined confidentiality safeguards and data usage protocols before administration. On-site questionnaires were completed within 15 min and collected immediately. Of the 600 returned surveys, data quality was assessed against three exclusion criteria—incomplete demographic information, patterned responses, and missing items. After excluding invalid surveys, 545 valid responses remained, corresponding to a 90.83% valid response rate. The sample included 289 males and 256 females, distributed across academic years as follows: 166 freshmen, 160 sophomores, 141 juniors, and 78 seniors. Participants’ ages ranged from 18 to 25 years (M = 20.49 ± 1.59) (Table 1). The research design and procedures are scientifically rigorous, equitable, and pose no harm or risk to participants. Recruitment adhered to the principles of voluntary, informed consent, with participants’ rights and privacy rigorously protected. The rights and privacy of participants were strictly safeguarded throughout all stages of the study. All procedures conformed to institutional ethical guidelines. No conflicts of interest or ethical and legal prohibitions are present in this project.

**Table 1:** Demographic characteristics of participants.

Causality	Form	Quantities	Percentage
Gender	Male student	289	53.03%
	Female student	256	46.97%
Age	18–22	387	71.01%
	23–25	158	28.99%
Grade	Freshman	166	30.46%
	Sophomore	160	29.36%
	Junior	141	25.87%
	Senior	78	14.31%

### 2.2 Measures

#### 2.2.1 Physical exercise

The Physical Activity Rating Scale-3 (PARS-3), as revised by Liang (1994), was used to assess physical exercise. The scale comprises three single-item dimensions—intensity, duration, and frequency—each rated on a Likert-type scale. Intensity and frequency are scored from 1 to 5, while duration is scored from 0 to 4. A composite exercise volume score is computed by multiplying the intensity, frequency, and duration scores, with higher totals indicating greater engagement. In the present sample, the PARS-3 demonstrated acceptable internal consistency (Cronbach’s *α* = 0.728).

#### 2.2.2 Perceived social support

Perceived social support was measured using the 12-item Perceived Social Support Scale (PSSS; Zimet et al., 1988), which evaluates subjective support across three domains: family, friends, and others. Items are rated on a 7-point Likert-type scale, with higher aggregate scores reflecting stronger perceived support. The PSSS exhibited excellent reliability in this study (Cronbach’s α = 0.926).

#### 2.2.3 Meaning in life

The Meaning in Life Questionnaire (MLQ; Steger et al., 2006; revised by Liu and Gan, 2010) was administered to assess existential meaning. This 9-item instrument comprises two subscales—presence of meaning and search for meaning—each scored on a 7-point Likert-type scale. Higher scores denote a stronger sense of life meaning. The MLQ demonstrated high internal consistency in the current sample (Cronbach’s α = 0.914).

#### 2.2.4 Suicidal ideation

Suicidal ideation was assessed with the 14-item Self-rating Idea of Suicide Scale (SIOSS; Fu and Zheng, 2009), encompassing three dimensions: suicidal desire, suicidal motivation, and suicidal planning. Items are rated on a 5-point Likert-type scale, with higher scores indicating more severe ideation. The SIOSS showed excellent reliability in this study (Cronbach’s α = 0.933).

### 2.3 Data analysis

All statistical analyses were conducted using SPSS 27.0. Descriptive statistics, reliability analyses, and Pearson correlations were performed in SPSS, while multiple regression, mediation, and moderation analyses were executed using Hayes’s PROCESS macro (Model 4 and Model 85) with bias-corrected percentile bootstrapping. Given the cross-sectional design, causal inferences cannot be drawn.

## 3 Results

### 3.1 Common method bias tests

Common method variance was evaluated via Harman’s single-factor test (Podsakoff et al., 2003). The unrotated factor solution yielded nine factors with eigenvalues greater than 1, and the first factor accounted for 32.62% of the total variance—below the 40% threshold—indicating that common method bias did not substantially influence the results.

### 3.2 Descriptive statistics and correlations

Table 2 presents Pearson correlation coefficients among physical exercise, perceived social support, meaning in life, and suicidal ideation. Physical exercise correlated positively with perceived social support (*r* = 0.370, *p* < 0.01) and meaning in life (*r* = 0.434, *p* < 0.01), and negatively with suicidal ideation (*r* = −0.296, *p* < 0.01). Perceived social support showed a strong positive association with meaning in life (*r* = 0.624, *p* < 0.01) and a negative association with suicidal ideation (*r* = −0.512, *p* < 0.01). Meaning in life was also inversely related to suicidal ideation (*r* = −0.493, *p* < 0.01).

**Table 2:** Descriptive statistics and correlations.

Variable	*M*	*SD*	1	2	3	4
1. Physical exercise	21.343	19.562	1			
2. Perceived social support	62.283	11.894	0.370***	1		
3. Meaning in life	41.189	11.141	0.434***	0.624***	1	
4. Suicidal ideation	16.087	9.933	−0.296***	−0.512***	−0.493***	1

*N* = 545. ***p < 0.01; all tests were two-tailed.

### 3.3 The chain mediating effects analysis

Significant associations emerged between physical exercise, perceived social support, meaning in life, and suicidal ideation across all pairwise comparisons, thereby satisfying the statistical assumptions required for sequential mediation analyses of perceived social support and meaning in life. Employing Model 6 of the PROCESS macro in SPSS (Hayes, 2012), this study adjusted for demographic covariates and evaluated the sequential mediation pathway using 5,000 bootstrap resamples with bias-corrected 95% confidence intervals.

The regression analysis results (Table 3) demonstrated that physical exercise significantly and negatively predicted college students’ suicidal ideation (*β* = −0.154, *p* < 0.01). When perceived social support and meaning in life were first incorporated into the regression model, physical exercise retained its significant negative predictive effect on suicidal ideation (*β* = −0.041, *p* < 0.05). Furthermore, physical exercise positively predicted perceived social support (*β* = 0.218, *p* < 0.01) and meaning in life (*β* = 0.131, *p* < 0.01), respectively. Perceived social support, in turn, positively predicted meaning in life (*β* = 0.513, *p* < 0.01) while negatively predicting suicidal ideation (*β* = −0.243, *p* < 0.01), and meaning in life also significantly negatively predicted suicidal ideation (*β* = −0.246, *p* < 0.01).

**Table 3:** Regression analysis of the relationship between physical exercise and suicidal ideation.

Outcome	Predictor	*R* ^2^	*F*	*β*	*t*	95% Boot CI
Suicidal ideation	Gender	0.134	20.979^***^	2.770	3.315^***^	[1.129,4.412]
	Age			−0.447	−0.967	[−1.353,0.460]
	Grade			1.900	2.691^***^	[0.513,3.287]
	Physical exercise			−0.154	−7.317^***^	[−0.195,–0.112]
Perceived social support	Gender	0.175	28.636^***^	−1.522	−1.557	[−3.442,0.398]
	Age			0.958	1.775^*^	[−0.102,2.019]
	Grade			−3.071	−3.719^***^	[−4.693,–1.449]
	Physical exercise			0.218	8.900^***^	[0.170,0.267]
Meaning in life	Gender	0.440	84.542^***^	0.718	0.949	[−0.769,2.205]
	Age			−0.239	−0.571	[−1.061,0.583]
	Grade			0.657	1.016	[−0.613,1.926]
	Physical exercise			0.131	6.456^***^	[0.091,0.171]
	Perceived social support			0.513	15.429^***^	[0.448,0.578]
Suicidal ideation	Gender	0.338	45.880^***^	2.385	3.248^***^	[0.942,3.827]
	Age			−0.151	−0.373	[−0.948,0.645]
	Grade			0.927	1.479	[−0.304,2.158]
	Physical exercise			−0.041	−1.992^**^	[−0.081,–0.001]
	Perceived social support			−0.243	−6.286^***^	[−0.319,–0.167]
	Meaning in life			−0.246	−5.885^***^	[−0.328,–0.164]

*N* = 545. Boot confidence interval (CI) upper limit (ULCI), and boot CI lower limit (LLCI) are indicative of the standard deviation (SD) of the indirect effect evaluated through the deviation correction percentile Bootstrap approach and the upper and lower limits of the respective 95% confidence intervals (CIs). CI, confidence interval. *p < 0.1; **p < 0.05; ***p < 0.01.

**Figure 2:**
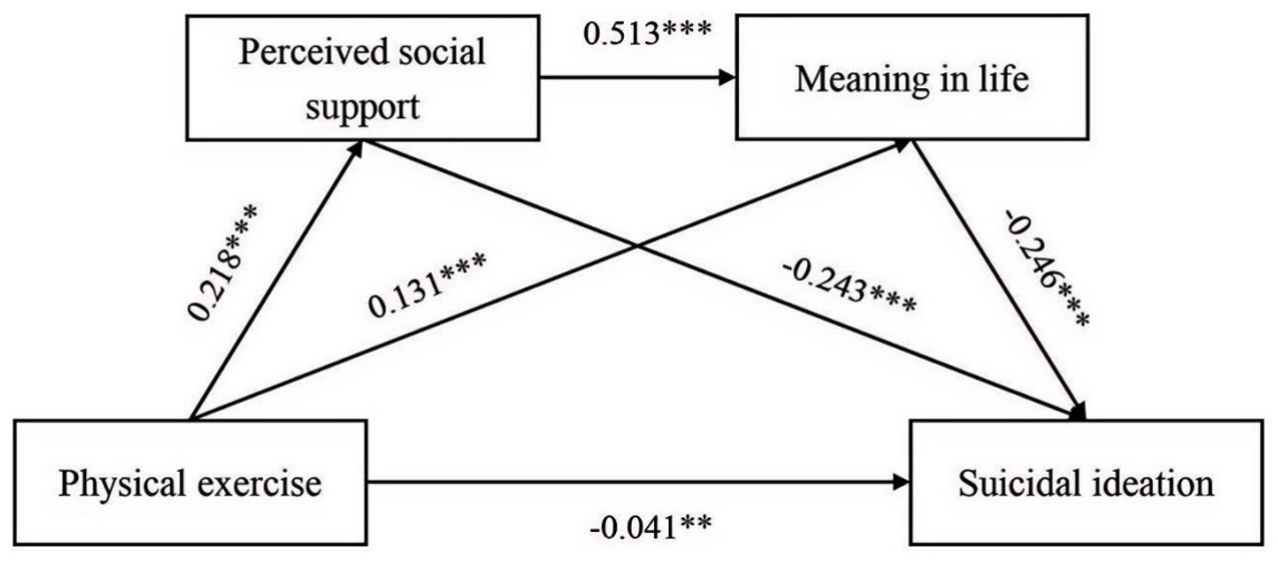
The chain mediation effect of perceived social support and meaning in life. **p < 0.05; ***p < 0.01.

Sequential mediation analysis (Table 4) indicated that perceived social support and meaning in life mediated the association between physical exercise and college students’ suicidal ideation, yielding a total indirect effect of −0.113. The 95% bias-corrected confidence interval (CI) [−0.138, −0.090] did not include zero, confirming the mediation, which accounted for 73.38% of the total effect of physical exercise on suicidal ideation (total effect: −0.154). Three indirect pathways were identified: (1) Indirect Effect 1 (mediating effect: −0.053) via the “physical exercise → perceived social support → suicidal ideation” pathway, contributing 34.42% of the total effect; (2) Indirect Effect 2 (mediating effect: −0.032) via the “physical exercise → meaning in life → suicidal ideation” pathway, accounting for 20.78% of the total effect; (3) Indirect Effect 3 (mediating effect: −0.028) via the sequential “physical exercise → perceived social support → meaning in life → suicidal ideation” pathway, representing 18.18% of the total effect. All CIs excluded zero, indicating statistically significant indirect mediation. The comprehensive path model depicting these relations is presented in Figure 2.

**Table 4:** Perceived social support and meaning in life in the mediation effect analysis.

Effect types	Indirect effects	Boot SE	Boot LLCI	Boot ULCI	Relative mediation effect
Total indirect effect	−0.113	0.013	−0.138	−0.090	73.38%
Indirect effect 1	−0.053	0.011	−0.076	−0.034	34.42%
Indirect effect 2	−0.032	0.008	−0.049	−0.017	20.78%
Indirect effect 3	−0.028	0.005	−0.038	−0.017	18.18%
Compare 1	−0.021	0.016	−0.052	0.009	
Compare 2	−0.026	0.013	−0.052	−0.002	
Compare 3	−0.005	0.008	−0.021	0.011	

*N* = 545. Boot SE, Boot LLCI, and Boot ULCI refer, respectively, to the standard error and the lower and upper limits of the 95% confidence interval of the indirect effects estimated by the percentile bootstrap method with deviation correction.

### 3.4 The moderation effects analysis

**Figure 3:**
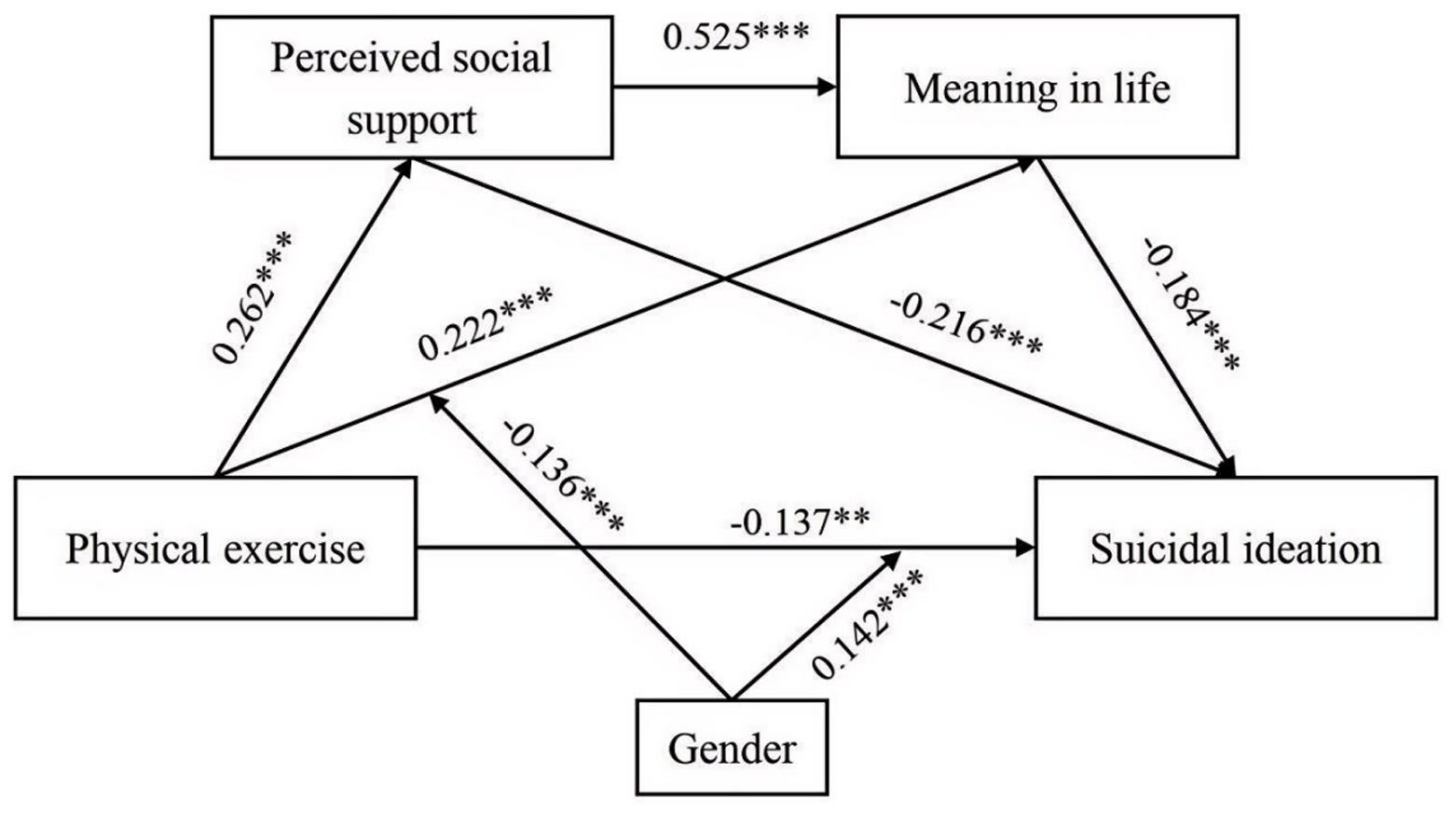
The model of moderated chain mediating effect. **p < 0.05; ***p < 0.01.

To investigate whether gender moderated the mediation pathways, we conducted moderated regression analyses using Model 92 of the PROCESS macro (Table 5). Incorporating gender into the model revealed that the physical exercise × gender interaction term significantly and negatively predicted meaning in life (*β* = −0.136, *p* < 0.01), indicating that gender moderated the initial stage of the mediation pathway from physical exercise to meaning in life to suicidal ideation (Figure 3). This finding suggests that physical exercise elicited stronger increases in meaning in life among female college students than among their male counterparts. In contrast, the physical exercise × gender interaction term significantly and positively predicted suicidal ideation (*β* = 0.142, *p* < 0.01), demonstrating that gender also moderated the direct effect of physical exercise on suicidal ideation. Specifically, the reduction in suicidal ideation associated with physical exercise was more pronounced among male college students relative to females.

**Table 5:** Test of moderated chain mediation.

Outcome	Predictor	*R* ^2^	*F*	*β*	*t*	95% Boot CI
Perceived social support	Physical exercise	0.147	30.983^***^	0.262	6.270^***^	[0.180,0.344]
	Gender			−1.425	−1.009	[−4.201,1.350]
	Physical exercise × Gender			−0.040	−0.781	[−0.142,0.061]
Meaning in life	Physical exercise	0.454	59.506^***^	0.222	6.624^***^	[0.156,0.287]
	Perceived social support			0.525	11.881^***^	[0.438,0.612]
	Gender			6.625	1.720^*^	[0.941,14.191]
	Physical exercise × Gender			−0.136	−3.264^***^	[−0.219,–0.054]
	Perceived social support × Gender			−0.496	−0.766	[−0.177,0.078]
Suicidal ideation	Physical exercise	0.348	40.927^***^	−0.137	−3.832^***^	[−0.207,–0.067]
	Perceived social support			−0.216	−3.918^***^	[−0.325,–0.108]
	Meaning in life			−0.184	−2.780^***^	[−0.313,–0.055]
	Gender			9.674	2.541^**^	[2.196,17.152]
	Physical exercise × Gender			0.142	3.259^***^	[0.056,0.227]
	Perceived social support × Gender			−0.109	−1.429	[−0.260,0.041]
	Meaning in life × Gender			−0.075	−0.876	[−0.243,0.093]

*N* = 545. Boot confidence interval (CI) upper limit (ULCI), and boot CI lower limit (LLCI) are indicative of the standard deviation (SD) of the indirect effect evaluated through the deviation correction percentile Bootstrap approach and the upper and lower limits of the respective 95% confidence intervals (CIs). CI, confidence interval. *p < 0.1; **p < 0.05; ***p < 0.01.

## 4 Discussion

### 4.1 The relationship between physical exercise and suicidal ideation

This study examined the association between physical exercise and suicidal ideation in college students. The findings indicate that physical exercise is a significant negative predictor of suicidal ideation, supporting Hypothesis H1 (Table 6) and corroborating earlier research (Brown et al., 2007). According to cognitive evaluation theory, motivation arises from both intrinsic and extrinsic sources, which are not mutually exclusive. External contingencies that control behavior may undermine self-determination, causing individuals to perceive their actions as externally regulated. In this regard, cognitive appraisals of environmental stimuli can modulate intrinsic motivation and, in turn, influence behavior (Song and Jin, 2024). In line with the affective benefits framework of exercise, physical activity exerts therapeutic effects on depression, anxiety, and stress (Liu, 2020). The stress–vulnerability model classifies determinants of suicidal ideation into protective and risk factors, where protective factors serve to buffer against suicidal thoughts (Abela and Skitch, 2015; Su et al., 2015). By alleviating symptoms of anxiety and depression, physical exercise may attenuate suicidal ideation. Moreover, exercise-induced upregulation of dopaminergic pathways has been shown to inhibit suicidal ideation (Roy et al., 1992). These results highlight the imperative for societal initiatives to emphasize physical exercise as a preventive measure against suicidal ideation in college populations, particularly by cultivating positive emotional experiences that enhance individuals’ sense of fulfillment through exercise.

**Table 6:** Verification results of research hypothesis.

Hypothesis	H1	H2	H3	H4	H5					
					H5a	H5b	H5c	H5d	H5e	H5f
Test results	Accept	Accept	Accept	Accept	Reject	Accept	Accept	Reject	Reject	Reject

### 4.2 The mediating effect of perceived social support

This study demonstrated that perceived social support significantly mediates the relationship between physical exercise and suicidal ideation in college students, thereby confirming Hypothesis 2 (Table 6). According to the social interaction hypothesis, interpersonal engagement during physical exercise fosters positive emotional states (Lu et al., 2024). In particular, spiritual, informational, or material support from exercise facilitators or peers constitutes a key mechanism for alleviating negative emotions and enhancing mood. Perceived social support mitigates stress and promotes positive affect, thereby improving students’ physiological and psychological well-being. Through exercise-related social interactions, college students form or strengthen their social networks, receiving support that bolsters their perceived ability to cope with stressful environments (Cohen et al., 2000) and provides tangible resources for stress management (Kawachi and Berkman, 2001). These results are consistent with the social support buffer model, which posits that social support attenuates risk factors such as adverse environments and negative affect, thus reducing suicide risk (Cohen and Wills, 1985). Prior research indicates that higher levels of social support weaken the adverse impact of stressful events on suicide risk (Lew et al., 2019), and that greater support is associated with lower reliance on maladaptive coping strategies, further diminishing vulnerability to suicide (Wen et al., 2024b). Consequently, strengthening perceived social support among college students should be a central component of suicide prevention efforts.

### 4.3 The mediating effect of meaning in life

This study revealed that meaning in life mediates the association between physical exercise and suicidal ideation in college students, thereby confirming Hypothesis 3 (Table 6). Prior evidence indicates that a health-promoting lifestyle correlates significantly and positively with meaning in life. As a core element of such a lifestyle, physical exercise also demonstrates a robust positive association with students’ perceived meaning in life (Ding et al., 2016). Moreover, engaging in regular physical activity promotes the secretion of endorphins, which fosters enhanced physical health and psychological well-being, elevates mood, and increases vitality among students; these benefits improve overall life satisfaction and indirectly strengthen one’s sense of meaning in life (Frankl, 1963). Meaning in life encompasses an individual’s cognitive awareness and understanding of human existence, its essence, and personally significant values (Steger et al., 2006). Research confirms that meaning in life buffers and moderates the deleterious effects of stressful life events, thereby reducing impulsive suicidal ideation or behaviors and effectively lowering suicide risk among adolescents (Kleiman and Beaver, 2013). Individuals who recognize their self-worth and possess a strong sense of self-efficacy and purposefulness tend to exhibit greater psychological resilience during crises, as their pursuit and attainment of meaning remain resilient to external disruptions. This further underscores the protective function of meaning in life against suicidal ideation in college students (Zhang et al., 2021). Although the mediating effect of meaning in life observed in this study is relatively modest in magnitude, such small effects may accumulate over time at the individual level. For example, college students who engage regularly in physical exercise may gradually develop a stronger sense of meaning and purpose in life. This shift could serve as a buffer in critical moments, potentially reducing the likelihood of emerging suicidal ideation.

### 4.4 The chain mediation effect of perceived social support and meaning in life

This study further demonstrated that perceived social support and meaning in life sequentially mediated the relationship between physical exercise and suicidal ideation among college students, thereby confirming Hypothesis 4 (Table 6). These results imply that although physical exercise serves as the foundational antecedent to suicidal ideation, the ensuing enhancement of perceived social support and meaning in life likely represents the critical mechanism through which exercise reduces suicidality. Social learning theory posits that behavioral outcomes shape subsequent behaviors not directly but via cognitive appraisal of experiential feedback (Guo and Zhang, 2022). In the wake of physical activity, students frequently report heightened well-being, increased achievement motivation, strengthened interpersonal bonds, and enriched social support networks (Morgan and Goldston, 2013). According to Fredrickson’s broaden-and-build theory of positive emotions, such positive subjective experiences foster enduring physiological, psychological, cognitive, and social resources, thereby deepening meaning in life and attenuating suicidal ideation (Fredrickson, 2001). Battista and Almond’s orientations to meaning framework emphasizes that a sense of meaningfulness emerges from healthy interpersonal connections and robust social services, underscoring social support’s pivotal role in cultivating existential purpose (Battista and Almond, 1973). From an intervention standpoint, even modest mediating effects hold practical significance: promoting physical exercise can enhance perceived social support and meaning in life, thus reducing suicidal ideation via multiple pathways and benefiting both individual mental health and the broader well-being of the student population.

### 4.5 The moderating role of gender

This study established that gender moderates both the initial stage of the “physical exercise → meaning in life → suicidal ideation” mediation pathway and the direct effect of physical exercise on suicidal ideation, thereby supporting Hypotheses 5b and 5c (Table 6). Regarding first-stage moderation, physical exercise constitutes a pivotal means of enhancing physical health and psychological resilience. Among female students, engagement in physical exercise not only improves physical fitness but also fosters self-identity formation, bolsters self-confidence, and cultivates social competencies (Zhou, 2024). As Yan and Wen (2025) demonstrated, physical exercise contributes significantly to advancing gender equality; accordingly, it more effectively augments meaning in life among female college students. Concerning the direct moderation effect, gender role socialization theory posits that males and females assume distinct roles and responsibilities during socialization, resulting in divergent behavioral patterns in physical exercise (Chen et al., 2022). Culturally prescribed gender norms grant males greater access to and engagement in physical activity, yielding more pronounced psychological benefits. This advantage is reflected in males’ adoption of more proactive coping strategies, with effect sizes markedly exceeding those observed in females. These findings underscore the imperative for gender-specific interventions that harness the positive psychological impacts of physical exercise to reduce suicidal ideation among college students.

### 4.6 Practical significance

This study elucidates the mechanisms by which physical exercise influences suicidal ideation among college students, thereby enriching theoretical frameworks of exercise-mediated suicide risk reduction and broadening intervention strategies to address student suicidality. The results offer both theoretical insights and practical guidance for developing exercise-based policies and suicide prevention measures. Specific implementation recommendations include:

First, design socially engaging physical activities. Universities could organize and promote team-based sports—such as basketball, soccer, and volleyball—to not only enhance students’ physical fitness but also foster interpersonal interaction, cooperation, and the strengthening of social support networks. Second, integrate meaning-enhancing activities. Encourage students to establish personal goals within physical exercise and pursue their attainment, thereby enhancing self-efficacy and a sense of accomplishment; for example, long-distance running challenges can provide participants with a clear objective and tangible achievement upon completion. Third, provide psychological support and resources by incorporating mental health education into physical education curricula, enabling students to acquire knowledge of mental health concepts and strategies to manage stress and negative emotions. For instance, tailored workshops can instruct students on using physical activity as a vehicle for stress relief and anxiety reduction. Fourth, cultivate a positive campus sports culture that promotes regular engagement in physical activity, thus strengthening social bonds and enriching meaning in life; high-profile events, such as campus sports festivals, can showcase student athletic achievements, thereby fostering collective pride and a sense of belonging.

Traditional Chinese cultural values profoundly influence the formation of social support and the sense of meaning in life. Confucian principles—emphasizing benevolence (ren) and relational harmony—encourage individuals to seek support primarily through close ties, such as family and friends. Simultaneously, the cultural emphasis on collective belonging and social obligations renders interpersonal physical activities—such as team sports and club involvement—particularly appealing, thereby effectively enhancing perceived social support. Moreover, within this collectivist context, college students often prioritize social contribution and external recognition; thus, incorporating elements such as volunteer service and community engagement into physical activities can more powerfully augment their sense of life meaning.

In summary, designing physical activity programs grounded in Chinese cultural values enables a more precise alignment with the psychological needs of college students. By leveraging exercise to reinforce social support and enhance life meaning, these culturally responsive strategies can effectively mitigate the risk of suicidal ideation. This approach affords campus mental health interventions locally adapted and potentially more efficacious solutions.

## 5 Limitations and prospects

In summary, this study examined the effects of physical exercise on college students’ suicidal ideation and its underlying pathways, offering critical insights for suicide prevention within this demographic. Nonetheless, several limitations merit consideration in subsequent investigations. First, the cross-sectional design restricts causal inference; future research should adopt experimental or longitudinal methodologies to verify causal linkages and employ objective activity measures (e.g., wearable devices) to quantify exercise engagement. Second, although this study addressed the mediating roles of perceived social support and meaning in life, it did not consider other influential variables—such as sleep disturbances, psychological resilience, and life satisfaction. Incorporating additional moderators or mediators would yield a more comprehensive understanding of these mechanisms. Finally, suicidality comprises distinct phenomena (e.g., non-suicidal self-injury, suicidal ideation, suicide attempts, and suicidal behaviors); comparative analyses of their shared and unique characteristics could refine theoretical models and empirical approaches to student suicidality, thereby informing more precisely targeted prevention strategies.

## 6 Conclusion

In this study, we examined the relationship between physical exercise and suicidal ideation among college students, focusing on the mediating roles of perceived social support and meaning in life. The findings reveal that physical exercise, perceived social support, and meaning in life each significantly and negatively predict suicidal ideation. Furthermore, perceived social support and meaning in life partially mediate the link between physical exercise and suicidal ideation, both independently and sequentially. Gender moderated the first half of the physical exercise → meaning in life → suicidal ideation pathway as well as the direct effect of exercise on ideation. These results clarify the mechanisms by which exercise and psychological factors influence suicidal ideation, confirm gender-specific differences in suicidal ideation among college students, provide empirical support for targeted mental health interventions to reduce suicide risk, and suggest new directions for intervention.

## Data Availability

The raw data supporting the conclusions of this article will be made available by the authors, without undue reservation.
